# Research Progress on Cu–15Ni–8Sn Alloys: The Effect of Microalloying and Heat Treatment on Microstructure and Properties

**DOI:** 10.3390/ma16175913

**Published:** 2023-08-29

**Authors:** Shaodan Yang, Kexing Song, Yanjun Zhou, Ran Yang, Yan Yu, Lele Liu, Jidong Chen, Fei Zhou, Wenhao Yang, Guoshang Zhang, Juan Du

**Affiliations:** 1 School of Material Science and Engineering, Zhengzhou University, Zhengzhou 450001, China; sdyang92@163.com; 2 Henan Academy of Sciences, Zhengzhou 450002, China; 3 School of Material Science and Engineering, Henan University of Science and Technology, Luoyang 471023, China; 4 School of Medical Technology and Engineering, Henan University of Science and Technology, Luoyang 471023, China; 5 Luoyang Ship Material Research Institute, Luoyang 471023, China; 6 Ningbo Boway Alloy Material Co., Ltd., Ningbo 315000, China

**Keywords:** Cu–15Ni–8Sn alloy, microalloying, preparation technology, heat treatment, microstructure, properties

## Abstract

Cu–15Ni–8Sn alloy is the best choice to replace beryllium bronze alloy. This alloy has unparalleled application value in aerospace, ocean engineering, electronic information, equipment manufacturing, and other fields. However, the application of Cu–15Ni–8Sn alloy is challenged and limited because of a series of problems in its preparation and processing, such as easy segregation, difficult deformation, and discontinuous precipitation. It is an effective way to improve the comprehensive properties of Cu–15Ni–8Sn alloy using alloying design and process optimization to control the as-cast, deformed, and heat-treated microstructures. At present, it is a hot spot for scholars to study. In this paper, the grade generation, system evolution, and preparation technology development of Cu–15Ni–8Sn alloy are comprehensively reviewed. The phase transformation sequence of the Cu–15Ni–8Sn alloy is discussed. The influence of the type, amount, and existing form of alloying elements on the strength of Cu–15Ni–8Sn alloy and its mechanism are systematically summarized. Furthermore, the latest research progress on the effects of solid solution, cold deformation, and aging on the phase structure transformation and mechanical properties of Cu–15Ni–8Sn alloy is summarized. Finally, the future development trend of the Cu–15Ni–8Sn alloy is projected. The research results of this paper can provide a reference for the control of the microstructure and properties of high-performance Cu–15Ni–8Sn alloys used in key fields, as well as the optimization of the preparation process and alloy composition.

## 1. Introduction

The Cu–15Ni–8Sn (C72900) alloy is a typical spinodal hardened copper alloy engineered for high strength, elasticity, and stress relaxation resistance. Compared with Cu-Be alloy, the comprehensive properties of Cu-15Ni-8Sn alloy are equal, and it has no toxicity during manufacturing and processing. In addition, the properties of Cu-15Ni-8Sn alloy are much better than those of Cu-Be alloy at high temperature. As the best substitute material for beryllium bronze, it has potential applications in electronic information, equipment manufacturing, and other fields, such as high-end connectors, instrumentation sensors, and other key components. In recent years, with the rapid development of aerospace, heavy-duty equipment, ocean engineering, and oil and gas exploitation, higher requirements have been put forward for the comprehensive properties of the Cu–15Ni–8Sn alloy. This alloy requires high toughness, wear resistance, and corrosion resistance while maintaining high strength. However, it is difficult to control these comprehensive properties due to the contradictory relationship among the above performances. Therefore, the comprehensive performance control of Cu–15Ni–8Sn alloy under multi-scene service conditions has become a hot and difficult topic for relevant scholars.

Previous research shows that there are a series of key technical and common scientific problems in the preparation and processing of Cu–15Ni–8Sn alloy, such as easy segregation, difficult deformation, and discontinuous precipitation. Using alloying design and process optimization to control the as-cast, deformed, and heat-treated microstructures is an effective way to improve the comprehensive properties of Cu–15Ni–8Sn alloy. In view of the above challenges, relevant scholars have carried out a series of studies and published some research findings. For example, researchers have tried different solutions to the segregation problem of the Cu–15Ni–8Sn alloy. Among them, the team at Central South University utilized powder metallurgy technology, the University of Science and Technology Beijing used L-PBF technology, and the Henan University of Science and Technology adopted rare earth microalloying and electromagnetic stirring. It is found that the above methods can improve the degree of segregation to a certain extent. For the thermal deformation process, scholars combine numerical simulation and physical experiments to optimize the thermal deformation process parameters and then control the deformed structure. For the heat treatment process, the relevant team studied the combined arrangement and process optimization of solid solution, cold deformation, and aging. It is found that by regulating the amplitude modulation decomposition process, controlling the dispersion distribution of precipitates, and inhibiting discontinuous precipitation, comprehensive performance, such as high strength and high elongation, can be improved.

In this paper, the development history, industrial status, typical application scenarios, and performance requirements of Cu–15Ni–8Sn alloy, as well as the development of preparation and processing technology, are summarized. According to the difficult problems in the industrial production process, the research progress on the influence of trace alloy elements and heat treatment processes on the alloy microstructure characteristics and comprehensive performance is discussed. Finally, the development trend of the Cu–15Ni–8Sn alloy is projected.

## 2. Development Process of Cu–15Ni–8Sn Alloy

In 1933, Eash J. T. et al. [1] improved the quality of the ingot and strength of the Cu-Ni-Sn alloy by adding Ni to the Cu-Sn alloy and found the aging hardening phenomenon of this alloy. In the 1960s, Hillert, Cahn, and Hilliard et al. [2,3] established the spinodal theory. In the 1970s, Schwartz L. H. et al. [4] observed that spinodal decomposition occurred in Cu-Ni-Sn alloy during aging by TEM. Thereafter, Plewes J. T. [5] applied a large cold deformation before the aging treatment of Cu-Ni-Sn alloy, which obviously improved the plasticity of the alloy and laid the foundation for the practical application of this alloy.

Bell Laboratories [6] exploited the Cu-Ni-Sn alloys in the late 1960s, developing innovations and patents in the 1970s. In the 1980s, the industrial production standard was formulated. Additionally, the Cu-Ni-Sn alloy brand of the American production technology standard mainly includes C72500 (Cu-9Ni-2Sn), CDA725 (Cu-9Ni-2.5Sn), C72600 (Cu-4Ni-4Sn), C72650 (Cu-7.5Ni-5Sn), C72700 (Cu-9Ni-6Sn), C72800 (Cu-10Ni-8Sn), C72900 (Cu–15Ni–8Sn), etc. [7]. Among them, the standard-specified components of C72900 (Cu–15Ni–8Sn) alloy are as follows: Ni 14.5–15.5 wt.%, Sn 7.5–8.5 wt.%, impurity Mn 0.05–0.3 wt.%, and impurity Fe and Zn shall not be more than 0.5 wt.% [7]. However, due to technical difficulties such as segregation and thermal processing cracking in the industrial preparation of Cu–15Ni–8Sn alloy, the commercial application of this alloy is greatly limited. In the 1980s, Pfizer Inc. produced Cu–15Ni–8Sn alloy strip and wire by using powder metallurgy technology [8], but the product size was too small to meet the needs of industrial applications. In the 1990s, Materion Brush Inc. [9] invented the EquaCast continuous casting technology and obtained a Cu–15Ni–8Sn ingot with a uniform solidification structure. This technology can effectively inhibit the segregation of Sn in the alloy and improve machinability. In addition, with the breakthrough of industrialization technology for Cu–15Ni–8Sn alloy, researchers pay more and more attention to the research of alloy application properties such as corrosion resistance, stress relaxation resistance, and wear resistance [10,11,12,13,14,15].

At present, Materion Inc. (Brush, United States) [9] has produced various Cu–15Ni–8Sn products with a yield strength range of 620–1380 MPa. The performance of the main products is shown in Table 1. Among them, ToughMet^®^3AT series products are mainly strengthened by spinodal decomposition. While maintaining high strength, the alloy has good wear resistance, high-temperature stress relaxation resistance, and corrosion resistance. The main types of application products are rods, pipes, plates, and rings. It can be used as wear-resistant and corrosion-resistant parts such as bearing bushings and valves in aerospace, heavy-duty equipment, oil and gas exploitation, and other fields. ToughMet^®^3TS is a high-strength and high-toughness alloy developed by the deformation heat treatment process. The hardening method is work hardening and spinodal decomposition, and the strength is obviously higher than that of the ToughMet^®^3AT series. This alloy has excellent resistance to dynamic impact loads and wear, especially in acidic environments or when exposed to seawater, and it also possesses non-magnetic and anti-knock properties. The main types of application products are rods, pipes, and wires, which can meet the performance requirements in harsh service environments in the field of oil and gas exploitation. BrushForm^®^158 series products can achieve the highest tensile strength of 1415 MPa by applying large cold deformation before aging, which meets the requirements of key components such as electronic connectors, switches, and sensors in the electronic industry. The application product type is strip.

The commercial Cu–15Ni–8Sn alloy currently developed by Lebronze Inc. is shown in Table 2, and its yield strength ranges from 620 MPa to 1069 MPa. This series of alloys focuses on key components such as sucker rods, valves, mud pumps, LWD, and MWD in oil and gas production [16]. In recent years, Concast Inc. and NGK Inc. have also launched Cu–15Ni–8Sn alloy series products for oil exploitation.

The research on Cu–15Ni–8Sn alloy in China began in the 1980s. Since the 21st century, a lot of research work has been carried out at Central South University, South China University of Technology, Dalian University of Technology, and Shanghai University of Technology. At present, in the industrial production of Cu–15Ni–8Sn alloy, Boway Inc., Albetter Inc., Shantou Huaxing Inc., Jinchuan Inc., and other enterprises have also carried out industrial small batch production and have made breakthroughs in some performance indexes of pipe rod.

## 3. Preparation Technology of Cu–15Ni–8Sn Alloy

The Cu–15Ni–8Sn alloy contains a large amount of Sn with a low melting point (231.89 °C), and the solidification temperature range of the solid–liquid phase is wide. Therefore, the macrosegregation and microdendrite segregation of Sn are easy to occur in the casting process, which leads to the difficulty of subsequent deformation and the decline of the comprehensive properties of the alloy. This is due to the rapid cooling rate of alloy melt during non-equilibrium solidification of liquid alloy. The melt volume shrinks, and negative pressure is generated in the ingot. In addition, the diffusion rates of Sn solute atoms in the liquid phase and solid phase are different, which leads to the redistribution of Sn solute atoms. Further, the Sn-rich melt moves to the ingot surface along the coarse dendritic gap. Finally, the phenomenon that the outer surface is rich in Sn but the center is poor in Sn is formed. The segregation of Sn will degrade the processability, strength, wear resistance, and corrosion resistance of the alloy. Therefore, controlling the segregation of the Sn element to obtain an alloy with uniform composition and microstructure is the premise for improving the comprehensive properties of the Cu–15Ni–8Sn alloy. At present, researchers have adopted rapid solidification, powder metallurgy, electromagnetic stirring, and other technologies to improve ingot quality.

### 3.1. Powder Metallurgy Technology

**Figure 1:**
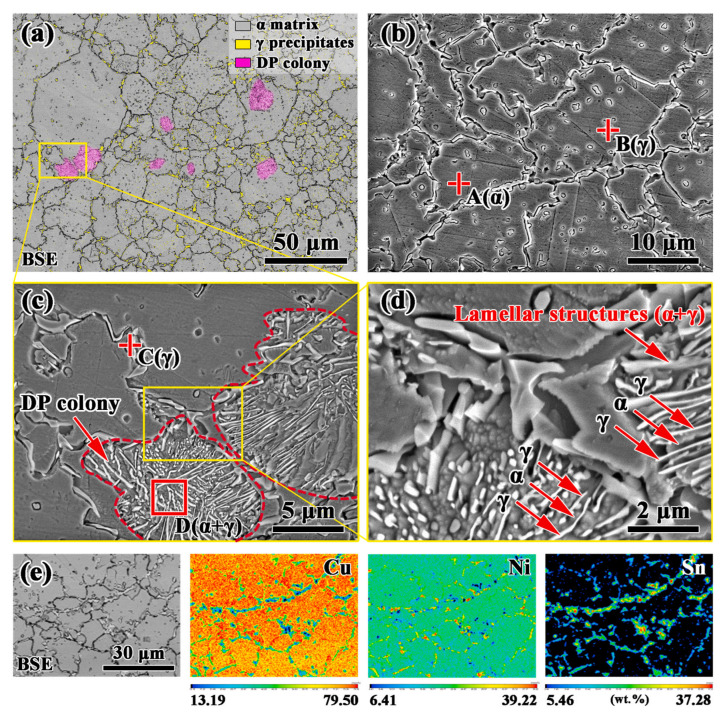
Micro-morphologies and element distributions of the HIP-fabricated Cu–15Ni–8Sn alloy: (**a**–**d**) SEM images at different magnifications; (**e**) EPMA mapping images for the Cu, Ni, and Sn elements, showing that Sn segregation was suppressed at the micron scale. The red cross in the figure is the mark composed of corresponding elements in different areas tested by SEM-EDS. (Reproduced with permission from Elsevier [17]).

In this technology, the pre-alloyed powder is prepared by the atomization method, and the prepared mixed powder is added from the hopper between vertical rolls. The powder is sucked into the roll gap by the friction between the powder and the rolls, and the pressure of the rolls makes the powder particles mechanically adhere. After compaction, sintering, winding, and a series of high-temperature annealing and cold rolling steps are carried out to improve the density. In the 1980s, Pfizer Inc. [8] adopted this technology to commercialize Cu–15Ni–8Sn alloy with strip and wire. At present, powder metallurgy methods include mechanical crushing, mechanical alloying, vapor deposition, ion sputtering, and so on. Sintering methods mainly include hot isostatic pressing and cold isostatic pressing. Li J. et al. [17] prepared Cu–15Ni–8Sn alloy by hot isostatic pressing (HIP) technology. The results showed that when the Cu–15Ni–8Sn alloy was prepared at 800 °C under 120 MPa at a heating rate of 5 °C/min, the segregation on the ingot micron scale was completely suppressed, as shown in Figure 1. OUYANG Y. et al. [18] prepared the Cu-15Ni-8 Sn-*X*Nb alloy with cold isostatic pressure instead of the HIP. The alloy powders were sealed in rubble molds by isostatic cool pressing under 200 MPa for 20 min. Then, the pressed ingots were vacuum-sintered at 850 °C and 1 × 10^−3^ Pa for 240 min. The results show that the segregation of Sn is significantly improved, and the grain size of the Cu–15Ni–8Sn–0.3Nb alloy was about 11 µm after aging for 60 min. However, powder metallurgy technology is not suitable for industrial production because of its complex production process, long process, high cost, and small product specifications [9].

### 3.2. Rapid Solidification Technology

The cooling rate of rapid solidification technology can reach 10^5^ K/s. The ultra-high cooling rate and large melt heat dissipation area make the Sn element too late to diffuse, thus effectively reducing the segregation degree and dendrite segregation spacing of Sn so as to obtain materials with uniform composition, microstructure, and properties. Commonly used rapid solidification technologies mainly include single-roll rotary casting, spray forming, and strip throwing technology [19]. Collins L. E. et al. [20] prepared C72900 alloy strips with a thickness of 20–120 μm by the rapid solidification method. The results show that the macrosegregation of the Sn element has been completely eliminated, but the microsegregation still exists. In the strip with a thickness of 70 μm, the greatest segregation was observed in the equiaxed region towards the outer surface. The Sn content varied from a minimum of 7.0 wt.% in the interior of grains to a maximum of about 10.5 wt.% at the grain boundaries. Deyong L. et al. [21,22] prepared Cu–15Ni–8Sn alloy samples without macrosegregation by the single-roll rotary casting rapid solidification method. Hermann P. et al. [23] obtained the Cu–15Ni–8Sn alloy by Osprey spray and found that Sn element segregation was inhibited and presented isoaxial crystals. However, rapid solidification technology is generally only suitable for making plates and strips with small cross-sectional dimensions [19].

### 3.3. Additive Manufacturing Technology

**Figure 2:**
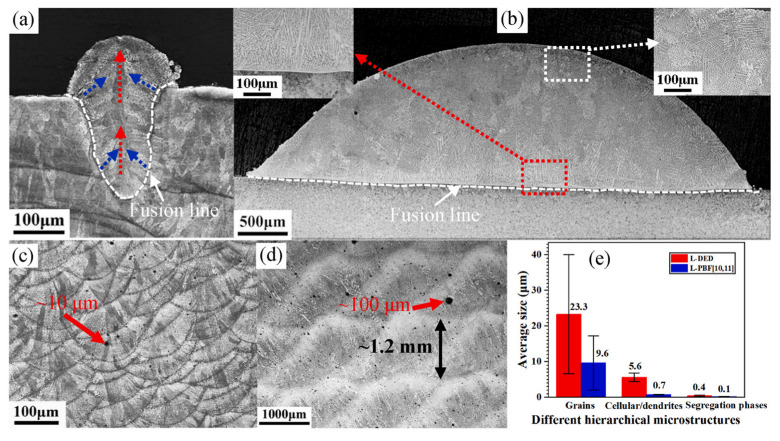
The cross-section optical micrographs of (**a**,**b**) single track and (**c**,**d**) multiple layers of LAM samples; images (**a**,**c**) are of L-PBF samples; images (**b**,**d**) are of L-DED samples; and (**e**) the comparison of different hierarchical microstructural sizes between L-DED and L-PBF samples. (Reproduced with permission from Elsevier [26]).

As a revolutionary industrial technology, additive manufacturing technology has attracted wide attention from scholars [24]. Specifically, in the machining process, a steep temperature gradient of up to ~10^7^ K/s and a high cooling rate of ~10^7^ K/m can be achieved during the additive manufacturing process [25], which shows excellent advantages in easy segregation and difficult machining of metals. At present, laser powder bed fusion (L-PBF, also known as SLM) and laser directional energy deposition (L-DED) are commonly used in the metal field. And it is found that L-PBF is more suitable than L-DED for preparing easily segregated Cu–15Ni–8Sn alloy [26,27,28,29,30]. Compared with Cu–15Ni–8Sn alloy blanks prepared by L-DED and L-PBF, the average size of tin-segregation phases of the L-DED (0.40 ± 0.20 μm) was three times higher than that of the L-PBF (0.10 ± 0.05 μm), as shown in Figure 2 [26]. The microstructures of the L-PBF-manufactured sample are mainly composed of epitaxially grown slender cellular structures with submicron widths. In contrast, the L-DED-manufactured sample is composed of fine dendrites with a width of 5.6 ± 1.2 μm [26]. Macrosegregation is not found in L-PBF-manufactured samples, but microsegregation was observed only under TEM. In L-PBF-manufactured samples, 80–200 nm Sn-enriched precipitates were dispersed between cellular structures [27]. The strengthening mechanism of L-PBF-manufactured alloy is mainly fine grain strengthening and dislocation strengthening caused by high dislocation density [28,29,30]. Currently, the preparation of Cu–15Ni–8Sn alloy by additive manufacturing technology has excellent potential and has become a research hotspot. However, a series of scientific problems existing in the laboratory stage of this technology still need to be further studied. Meanwhile, this technology still faces a series of future challenges in the industrial preparation of alloys, such as the density of large-sized billets, comprehensive performance control, quality stability, and production efficiency.

### 3.4. Advanced Foundry Technology

**Figure 3:**
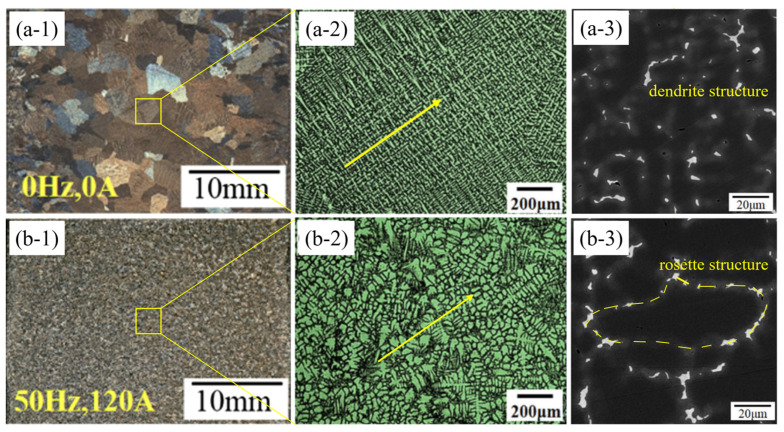
Images of the longitudinal section with and without EMS at different magnifications (**a-1**–**a-3**) 0 Hz, 0 A; (**b-1**–**b-3**) 50 Hz, 120 A. (Reproduced with permission from Springer [31]).

Most of the above preparation technologies are suitable for laboratory research or small-size billet production, and researchers have successively developed Cu–15Ni–8Sn alloy preparation technologies suitable for industrial production. For example, EquaCast continuous casting technology, invented by Materion Inc. [9], can obtain large-sized ingots with uniform structures. At present, the maximum ingot diameter can reach 635 mm, and the single weight is 15 t. Shanghai University [31] applies electromagnetic stirring (EMS) on the basis of horizontal continuous casting technology. The results show that the application of EMS is beneficial for grain refinement and for microstructure transformation from the dendrite to the rosette structure. It also leads to a significant improvement in the tensile property. The forced flow induced by EMS homogenizes the temperature field ahead of the solid–liquid interface, disturbing the heat flow direction and resulting in the columnar to equiaxed transition. The grain refinement under different electromagnetic stirring frequencies is mainly derived from the homogenization of the temperature and the remelting of dendritic arms, as shown in Figure 3. Dalian University of Technology [32,33] prepared Cu–15Ni–8Sn alloy ingot by vertical semi-continuous casting and applying mechanical vibration (MV) and electromagnetic field at the same time. It is found that when the amplitude of MV was 2 mm, the frequency was 57 time/min, the magnetic field power was 10 kW, the size of equiaxed grain on the whole cross-section of the ingot decreased from 2.24 mm to 0.94 mm, and the segregation rates of surface inverse segregation and center positive segregation improved from 11.5% and −12.6% to 2.1% and −1.4%.

## 4. Phase Transition Sequence and Microalloying of Cu–15Ni–8Sn Alloy

### 4.1. Phase Transition Sequence

**Figure 4:**
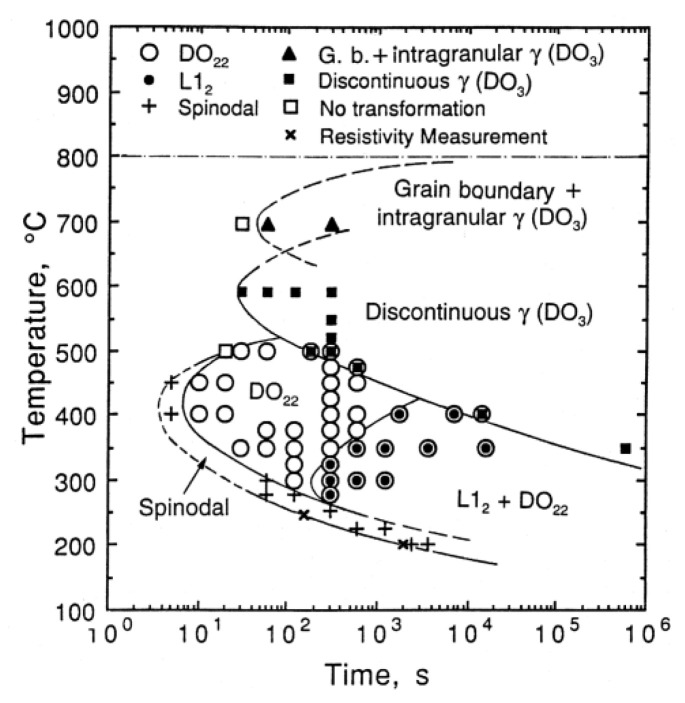
The TTT curve of Cu–15Ni–8Sn alloy. (Reproduced with permission from Elsevier [21]).

**Figure 5:**
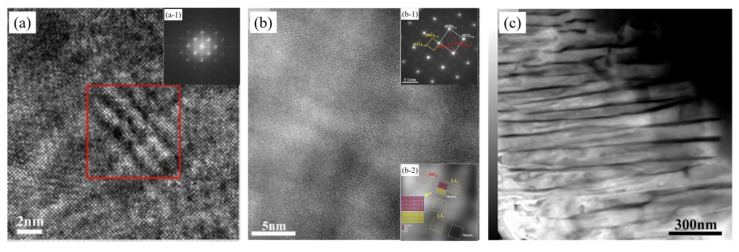
HRTEM–STEM images of phase transformation products at different aging stages of Cu–15Ni–8Sn–0.2Nb alloy. (**a**) HRTEM Image of aged at 400 °C for 2.5 min: spinodal structure; (**a-1**) corresponding FFT diffraction pattern; (**b**) HAADF-STEM image at atomic scale along [001] direction of aged at 400 °C for 90 min: DO_22_ and L1_2_; (**b-1**) SADP along [001] direction; (**b-2**) corresponding filtered IFFT image; (**c**) aged at 400 °C for 150 min: DP. (Reproduced with permission from Elsevier [35]).

After melting casting and homogenizing annealing, the solidification structure of the Cu–15Ni–8Sn alloy is obtained, and the segregation is obviously improved. Then, the alloy was subjected to thermomechanical treatment after hot deformation. Under the combined control of the solid solution, cold deformation, and aging, the alloy will successively undergo spinodal decomposition, ordering, discontinuous precipitation (DP), and continuous precipitation. The related microstructure characteristics and phase transformation sequence evolve simultaneously, which affects the comprehensive properties of the alloy. A lot of research on the phase transition process of the Cu–15Ni–8Sn alloy has been carried out before. Zhao J. C. et al. [34] studied the phase transformation process of Cu–15Ni–8Sn alloy by TEM and obtained the TTT curve, as shown in Figure 4. Studies have shown that the phase transformation products of Cu–15Ni–8Sn alloy during the aging process mainly include spinodal structure, DO_22_ ordered phase, L1_2_ ordered phase, discontinuous precipitated phase (DO_3_), and continuous precipitated γ phase at the grain boundary and intragranular [35], as shown in Figure 5. A large number of studies have shown that the time of spinodal decomposition is very short and may even have occurred in the solution quenching process [6,34,36]. The spinodal decomposition criterion is usually based on its structure diffraction reaction, such as the sideband appearing in the X-ray diffraction and the satellite spots appearing in the selected electron diffraction. However, because the spinodal decomposition process is too short, its decomposition process has not been dynamically observed so far. The mechanism of its occurrence and its influence on the subsequent phase transition process still need to be further explored. In addition, the formation mechanism and evolution process of the DO_22_ phase and L1_2_ phase in the ordering stage and the influence mechanism of interaction on the subsequent performance are still unclear. Wang Y. [36] found that DO_22_ and L1_2_ ordered structural formulas of Cu–15Ni–8Sn alloys can be expressed as (Cu*_x_*, Ni_1−*x*_)_3_Sn, where *x* values are different. At the same time, the related research shows that the formation of a discontinuous precipitate phase (DO_3_) in Cu–15Ni–8Sn alloy will seriously deteriorate the properties of the alloy. Still, the formation mechanism of the discontinuous precipitate phase and its influence on its properties need to be further explored [34]. In summary, there are many phase transformation processes in the heat treatment process of Cu–15Ni–8Sn alloy, and the interaction mechanism of microstructure characteristics is complex. Control of the comprehensive properties of this alloy through microstructure is the main goal of developing the alloy, and microalloying and heat treatment process control are effective ways to achieve this target.

### 4.2. Effect of Microalloying on Properties of Cu–15Ni–8Sn Alloy

**Figure 6:**
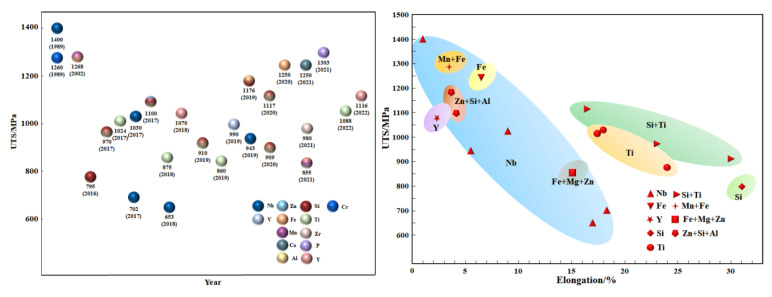
Development of microalloying design and its influence on properties of Cu–15Ni–8Sn alloy, data from [37,38,39,40,41,42,43,44,45,46,47,48,49,50,51,52,53,54,55,56].

By adding appropriate trace alloying elements to Cu–15Ni–8Sn alloy, the segregation can be effectively improved, and the amplitude modulation decomposition process can be affected. In addition, the discontinuous precipitation can also be inhibited, thus affecting the comprehensive performance of the alloy. At present, scholars have studied the influence of Nb [37,38,39,40], V [41], B [42], Mo [43], Fe [44], Co [45], Mg [46], Si [36], Ti [47,48,49,50], Zr [51], P [52], Y [53], and other elements on Cu–15Ni–8Sn alloy, mainly focusing on its microstructure features, phase change sequence, and properties. According to the different interaction modes between microalloying elements and matrix, it can be divided into insoluble metal type, alternative type, compound type, and multi-element additive type. The development of microalloying design and its influence on the properties of Cu–15Ni–8Sn alloy are shown in Figure 6 and Table 3.

#### 4.2.1. Insoluble Metal Type

Nb, V, B, and Mo belong to high-melting-point trace elements. The melting points of Nb, V, B, and Mo are 2468 °C, 1902 °C, 2300 °C, and 2617 °C, respectively, which are much higher than the melting points of Cu at 1084.6 °C. The combination of high melting points V and Ni forms a stable second phase with high melting points at grain boundaries and grains, which inhibits the nucleation and growth of discontinuous precipitates (DP). Nb can promote the spinodal decomposition process and significantly inhibit grain growth and discontinuous precipitation formation. Mo can improve the comprehensive properties of the Cu–15Ni–8Sn alloy by inhibiting the formation of discontinuous precipitation.

**Nb**: As can be seen from Table 3, when the additional amount of Nb is 0.2–0.3 wt.%, the tensile strength can reach 650–1400 MPa, and the elongation is 1–18%. Jiang B. et al. [37] prepared Cu–15Ni–8Sn–0.2Nb alloy by vacuum induction furnace smelting. The tensile strength of the alloy after aging is up to 1400 MPa, and the stress relaxation performance at 250 °C is better than beryllium copper, which is suitable for high-temperature elastic materials. The results show that Nb promotes the kinetics of spinodal decomposition of Cu–15Ni–8Sn. It not only accelerates its strengthening at the initial stage of spinodal decomposition but also promotes the growth of precipitates at grain boundaries [38]. The properties of alloys obtained by different preparation methods are also different. OUYANG Y. et al. [18] prepared Cu–15Ni–8Sn–0.3Nb alloy rods by means of powder metallurgy followed by hot extrusion, and it exhibited both higher strength (ultimate tensile strength > 1030 MPa) and higher tensile ductility (elongation > 9.1%) than Cu–15Ni–8Sn alloy after aging treatment. This study found that the improvement was caused by Nb-rich phases at grain boundaries, which led to the refinement of grain size and postponed the growth of discontinuous precipitates during aging. Wang N. et al. [35] think that the trace addition of Nb does not affect the precipitation type and sequence of Cu–15Ni–8Sn–0.3Nb.

**Figure 7:**
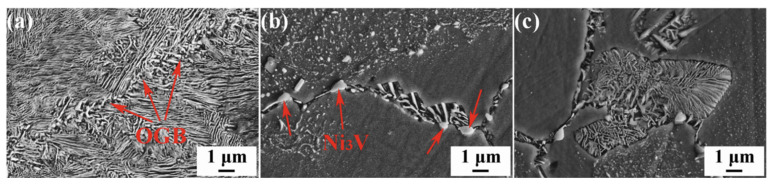
SEM images of discontinuous precipitation at grain boundaries for Cu–15Ni–8Sn–*x*V alloys aged at 400 °C for 240 min: (**a**) x = 0; (**b**) x = 0.4; (**c**) x = 1.0. (Reproduced with permission from Elsevier [41]).

V: Guo Z. et al. [41] studied the effects of different V addition amounts on the microstructure and properties of Cu–15Ni–8Sn alloy. The results showed that when the V content increased from 0 wt.% to 1.0 wt.%, the as-cast grain size of Cu–15Ni–8Sn–*x*V alloy decreased from 762 μm to 30 μm. When V content is 0.4 wt.%, the tensile strength, elastic modulus, and conductivity of Cu–15Ni–8Sn–0.4V alloy are 990 MPa, 131 GPa, and 8.0% IACS after aging at 400 °C for 240 min, respectively. The morphology and growth kinetics of discontinuous precipitation in Cu–15Ni–8Sn–*x*V alloy were studied experimentally. It was found that the DP reaction was controlled by grain boundary diffusion. In the Cu–15Ni–8Sn–0.4V alloy, the growth of DP was inhibited by the pinning effect of Ni_3_V particles on the grain boundary. However, when the V content is 1.0 wt.%, the alloy DP reaction can be accelerated, as shown in Figure 7.

B: The effects of B content (0.04, 0.08, 0.1, 0.3, and 0.5 wt.%) on the properties of Cu–15Ni–8Sn alloy were studied by Liu, S. et al. [42]. The results show that the Ni_6.67_SnB_2_ phase will be formed in an as-cast microstructure with the addition of B, and the segregation of the Sn element at the grain boundary could be alleviated to a certain degree. The hardness of Cu–15Ni–8Sn–0.1B alloy is 379 HV after aging at 400 °C for 360 min, which is slightly higher than that of Cu–15Ni–8Sn–0.04B and Cu–15Ni–8Sn–0.08B alloys. The appropriate B addition (0.04, 0.08, 0.1 wt.%) can greatly suppress the discontinuous precipitation behavior, while the excess B addition (0.3 and 0.5 wt.%) instead promotes the discontinuous precipitation behavior.

Mo: Zhang K. et al. [43] studied the effect of 0–1.5 wt.% Mo on the properties of Cu–15Ni–8Sn alloy. It was found that when the Mo content was 0.9 wt.%, the hardness of the alloy reached 384 HV. Further, Mo has a weak effect on improving as-cast microstructure segregation. In the stage of solid solution, when the content of Mo is lower than 0.3 wt.%, Mo dissolves in the matrix. When the content of Mo is higher than 0.9 wt.%, Mo exists as the second phase. In the stage of aging, the addition of proper Mo can significantly inhibit the nucleation and slow down the growth of DP. Excessive Mo content (greater than 0.9 wt.%) promotes the nucleation and growth of DP.

#### 4.2.2. Alternative Type

The alternative types of trace elements include Zn, Fe, Mn, Co, Al, Mg, etc. Zn, Fe, and Mn can be used as substitute elements for Cu, which can reduce the cost of the alloy and improve its processability. Co is near Ni in the Periodic Table of elements, and the chemical and physical properties are similar, so Co can be used as a substitute element for Ni. The addition of a small amount of Al and Mg can replace Sn, which is comparable to the high Sn content alloy in performance while reducing the cost.

**Figure 8:**
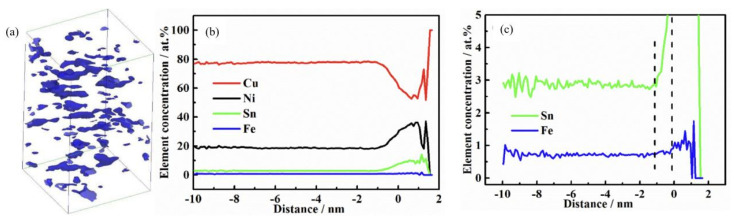
(**a**) Three-dimensional atom-probe atomic reconstruction of cold-rolled 0.5% Fe alloy during isothermal aging treatment at 400 °C for 60 min. (Reproduced with permission from Elsevier [44]); (**b**,**c**) Proxigram showing variation in composition as a function of distance from a 4.8 at.% Sn iso-concentration surface in a cold-rolled 0.5% Fe alloy during isothermal aging treatment at 400 °C for 60 min.

Fe: Guo C. et al. [44] investigated the effect of the addition of Fe at 0–0.5 wt.% on the properties of the Cu–15Ni–8Sn alloy. It was found that with the Fe content of 0.1 wt.%, the alloy hardness, tensile strength, and yield strength were 458 HV, 1250 MPa, and 960 MPa, respectively, which were 30 HV, 123 MPa, and 58 MPa higher than those without Fe, respectively. This paper used the 3D atomic probe to study the mechanism of Fe and found that Fe is mainly enriched in the core of the ordered phase, as shown in Figure 8. It was shown that the addition of Fe improved the DO_22_-ordered phase stability and inhibited the DP growth rate.

Co: Guo C. et al. [45] studied the effect of 0–1.5 wt.% Co content on the microstructure and properties of Cu–15Ni–8Sn alloy. It shows that when the addition of Co is 0.5 wt.%, the tensile strength (yield strength) and elastic modulus at peak aging are 1250 MPa (1197 MPa) and 149.8 GPa, respectively. The addition of trace Co elements can inhibit the precipitation and growth of discontinuous precipitation. Co enrichment is found in the γ-DO_3_ phase, which can change the interlamellar spacing, thus affecting the hardness of the alloy.

#### 4.2.3. Compound Type

Compound trace elements mainly include Si, Ti, Zr, P, Y, etc. These elements mainly form compounds with Ni and precipitate at grain boundaries or within grains, thus inhibiting the formation and development of discontinuous precipitates. Under suitable heat treatment conditions, the strength of alloys can be obviously improved. In recent years, there has been much research on this kind of alloy element, and the representative teams include Central South University, South China University of Technology, Jiangxi University of Science and Technology, and so on.

Si: Central South University [36] has carried out systematic research on Cu-Ni-Sn since the beginning of the 21st century. The research shows that the optimal addition amount of Si in Cu–15Ni–8Sn alloy is 0.3 wt.%, and the hardness can reach 457 HV. It was found that the addition of the Si element combined with Ni to form Ni_3l_Si_l2_ and Ni_3_Si, in which the Ni_3_Si phase has the same structure and size as the matrix α phase and has an obvious refinement effect on dendrites. Ni_3_Si can be reversibly dissolved and precipitated in the alloy with a change in temperature. It is easy to precipitate at grain boundaries during aging, occupying the nucleation position of γ phase discontinuous precipitation and inhibiting discontinuous precipitation in the later aging period. This inhibitory effect was strongest at 0.3 wt.% Si content. The increase in Si content made it easy to form insoluble Ni_3l_Si_l2_, but it also provided the nucleation position for discontinuous precipitation and promoted its growth.

Ti: Dalian University of Technology [47] studied the comprehensive properties of Cu–15Ni–8Sn alloy with 0–0.7 wt.% Ti. The hardness and tensile strength of Cu–15Ni–8Sn–0.2Ti alloy are 378.2 HV and 860.36 MPa, respectively. The effect of 0–0.5 wt.% Ti on the microstructure and properties of Cu–15Ni–8Sn alloy was studied at the South China University of Technology [48,49,50]. The optimum content of Ti was 0.3 wt.%. Compared with the alloy without Ti, the tensile strength increased from 953 MPa to 1024 MPa, and the elongation increased from 2.7% to 17.9%. The experimental results show that the addition of Ti forms the Ni_3_Ti phase during solidification, and the Ni_3_Ti phase has a pinning effect on grain boundary migration during solution treatment, which leads to grain refinement. In addition, the Ni_3_Ti phase at the grain boundary occupies the nucleation position of discontinuous precipitation, which inhibits discontinuous precipitation.

**Figure 9:**
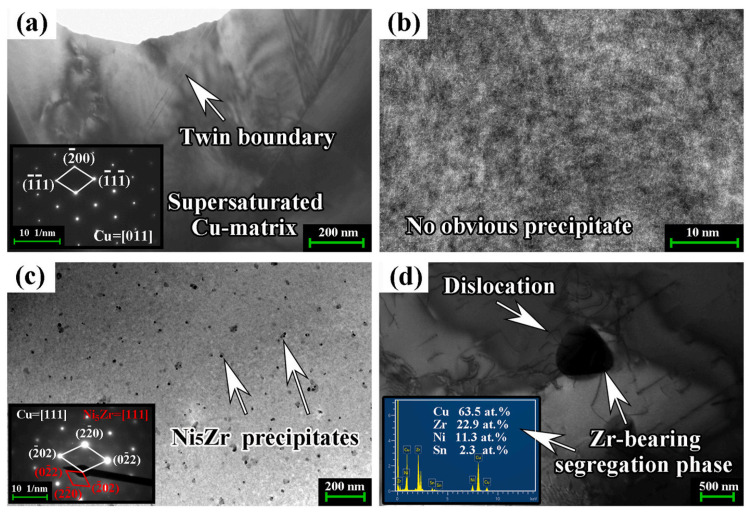
TEM images of as-solution treated Cu–15Ni–8Sn–*x*Zr alloys with different Zr contents: (**a**,**b**) 0.1 wt.%; (**c**) 0.3 wt.%; and (**d**) 0.5 wt.%. (Reproduced with permission from Elsevier [51]).

Zr: Zhang J. et al. [51] added 0–0.7 wt.% Zr to Cu–15Ni–8Sn. The results show that the hardness of the alloy is 330 HB, and the tensile strength is 980 MPa when the addition of Zr is 0.5 wt.%. Zr can reduce the segregation of Sn and change the precipitation morphology of the γ phase from the supersaturated α phase (flake → needle). When the content of Zr is lower than 0.1 wt.%, it mainly exists in the matrix as a solid solution atom. When the content increases to more than 0.3 wt.%, the existing form changes into a nano-scale precipitation phase (Ni_5_Zr) and then into a micron-scale segregation phase (Ni_4_SnZr), as shown in Figure 9. During aging, Zr can inhibit the nucleation and growth of DP significantly but cannot inhibit the growth of favorable ordered phases. When Zr content reaches 0.3 wt.%, DP content is reduced after aging for 16 h.

P: The effect of 0–0.3 wt.% P addition on the microstructure and properties of Cu–15Ni–8Sn alloy was studied by Jiangxi University of Technology [52]. The test results showed that when P content was 0.2 wt.%, the tensile strength (yield strength) and elastic modulus at peak aging were 1303 MPa (1280 MPa) and 145.0 GPa, respectively. The results show that the coarsening activation energy of Cu–15Ni–8Sn–0.2P alloy is 198.61 kJ/mol, which is less than 148 kJ/mol of Cu–15Ni–8Sn alloy. Based on the JMAK (Johnsone–Mehl–Avramie–Kolmogorov) equation, the diffusion activation energy Q of the discontinuous precipitation reaction of Cu–15Ni–8Sn–0.2P alloy is 221.90 kJ/mol, which is much higher than 105.45 kJ/mol of Cu–15Ni–8Sn alloy. The inhibitory effect of P addition on discontinuous precipitation is related to the distribution of the Ni_10_SnP_3_ phase, the formation of a non-precipitation zone, the decrease in grain boundary diffusion coefficient, and the increase in discontinuous precipitation layer spacing.

Y: Cu–15Ni–8Sn–0.2Y alloy was prepared by Cheng J. et al. [53]. The hardness of the peak-aging alloy was 372.2 HV, the yield strength was 1098 MPa, and the ultimate tensile strength was 1166 MPa. NiSnY and Ni_2_Y phases can be formed by adding Y elements to combine with Ni and Sn atoms, which are mainly distributed at grain boundaries and inhibit discontinuous precipitation. The strengthening effect of Y is mainly grain boundary strengthening and precipitation strengthening. After aging at 400 °C for 150 min, the grain size of Cu-15Ni-8Sn alloy increased by 10.08 µm, while that of Cu-15Ni-8Sn-0.2Y alloy only increased by 2.13 µm. 

#### 4.2.4. Multi-Element Additive Type

The composite type is composed of multiple microalloying elements, which has a significant effect on the microstructure and properties of the Cu–15Ni–8Sn alloy. In recent years, compound research has been mainly carried out around the above-mentioned substitutive and compound trace elements. The effects of 0–0.3 wt.% Si and 0–0.5 wt.% Ti on the microstructure and properties of Cu–15Ni–8Sn alloys were studied by the South China University of Technology [50]. The results showed that the tensile strength and yield strength of the alloys were 909 MPa and 708 MPa, respectively. The elongation was 29.6% when the content of Si and Ti was 0.3 wt.% and 0.1 wt.%, respectively. The Cu–15Ni–8Sn–0.25Mn–0.1Fe alloy developed by Ye J. [54] can reach 33 HRC at peak aging, and the tensile strength and elongation can reach 1268 MPa and 3.6%, respectively. Cu–15Ni–8Sn–1.0Zn–0.5Al–0.2Si alloy developed by Jiang Y. et al. [55] has a peak aging tensile strength of 1176 MPa, a yield strength of 1106 MPa, and an elongation of 3.86%. The tensile strength, elongation, and microhardness of Cu–15Ni–8Sn–0.14Fe–0.11Mn–0.22Zn developed by Xie et al. [56] are 855 ± 41 MPa, 15.2 ± 1.0%, and 292 ± 5 HV, respectively.

### 4.3. Heat Treatment of Cu–15Ni–8Sn Alloy

**Figure 10:**
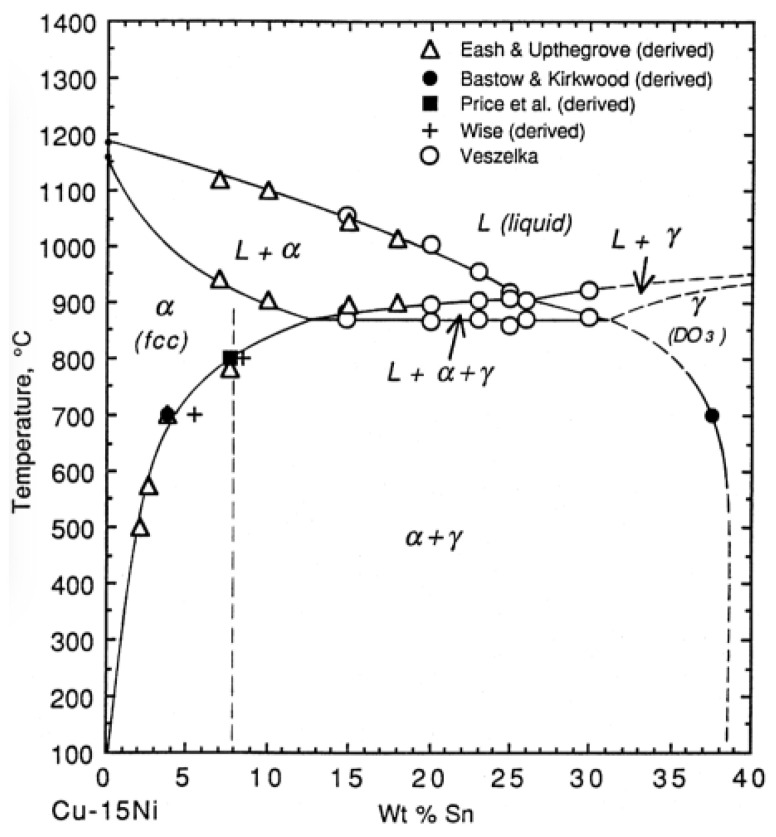
Pseudo binary phase diagram of (Cu-15Ni)-*x*Sn alloy. (Reproduced with permission from Elsevier [34]).

Based on the above-mentioned research review and analysis at home and abroad, in the composition optimization design stage of Cu–15Ni–8Sn alloy, different microalloying elements can improve segregation, refine grains, and affect the subsequent aging precipitation process. Furthermore, in the heat treatment process of the alloy, through the control of different heat treatment processes, the role of microalloying can be further exerted to the maximum extent. For example, it affects the decomposition process of amplitude modulation, promotes the characteristic evolution of precipitates during aging, inhibits discontinuous precipitation, etc. The properties of the Cu–15Ni–8Sn alloy are required for different application fields. According to the phase diagram of the (Cu–15Ni)–xSn alloy (Figure 10) [5,34], we can take full advantage of the synergistic strengthening effect of microalloying and heat treatment by optimizing the process parameters such as solution, cold deformation, and aging. Finally, we can realize the coordinated control of the comprehensive properties of Cu–15Ni–8Sn alloys, such as high strength, high toughness, high wear resistance, and high corrosion resistance. At present, the influence of the heat treatment process on the microstructure and properties of the Cu–15Ni–8Sn alloy has become the focus and hot spot. The comparison of properties of Cu–15Ni–8Sn alloys after heat treatment by different processes is shown in Table 4.

#### 4.3.1. Solid Solution + Aging

**Figure 11:**
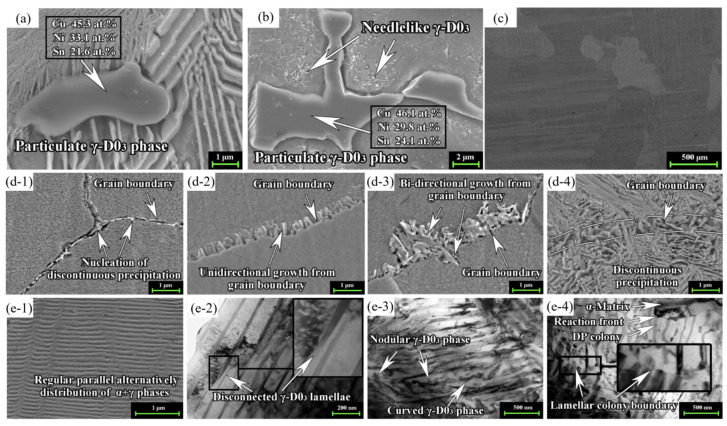
The γ-DO_3_ phase evolution of Cu–15Ni–8Sn alloy. (Reproduced with permission from Elsevier 57). (**a**) Particulate γ-DO_3_ phase in the solidified state; (**b**) particulate and a small amount of needlelike γ-DO_3_ phase in as-cast; (**c**) complete dissolution in solid solution state; (**d-1**–**d-4**) nucleation and growth processes of γ-DO_3_ phase during aging: (**d-1**) nucleation; (**d-2**) unidirectional growth; (**d-3**) multidirectional growth; (**d-4**) grain boundary coarsening; (**e-1**–**e-4**) different morphologies of γ-DO_3_ phase formed during aging: (**e-1**) equilibrium growth region; (**e-2**) disconnectedγ-D0_3_ phase; (**e-3**) disordered distribution region; (**e-4**) layered colony boundary. (Reproduced with permission from Elsevier [57]).

Zhang J. et al. [57] studied the effect of direct aging after homogenization annealing on the microstructure of Cu–15Ni–8Sn alloy. The tensile strength of this alloy can reach 890 MPa after aging at 400 °C for 240 min. The evolution rule of γ-DO_3_ phase characteristics at different aging times is found to be: particulate in the solidified state (Figure 11a) → particulate and a small amount of needlelike in as-cast (Figure 11b) → complete dissolution in the solid solution state (Figure 11c) → nucleation at the grain boundary and intracrystalline growth in the aging state (Figure 11d) → discontinuous precipitation (Figure 11e). Louzon T. J. et al. [58] studied the effect of a two-phase solution (675–775 °C) on the microstructure and properties of a Cu–15Ni–8Sn alloy. The results show that the yield strength of the alloy after peak aging treatment can reach 889 MPa, which is 55 MPa higher than that of a single-phase solution. The increase in strength is due to grain refinement caused by solid solution in the two-phase region, which delays the nucleation and growth of discontinuous precipitation at grain boundaries. Also, the alloys’ transformation kinetics were greatly slowed by the two-phase heat treatment. It is suggested that the slow kinetics of transformation observed are caused by grain size effects and grain boundary modifications. For example, the grain size is 40 μm after the solid solution at 825 °C for 30 min in the single-phase region and 3 μm after the solid solution at 725 °C for 60 min in the two-phase region. Zhao C. et al. [50] studied the effects of solution temperatures (800 °C, 820 °C, and 840 °C) on the microstructure and properties of Cu–15Ni–8Sn–0.3Si–0.1Ti alloy. The results show that no second phase is found in Cu–15Ni–8Sn alloy after solution at 820 °C for 60 min, but a Ni_16_Si_7_Ti_6_ second phase with an average size of about 184 nm exists in Cu–15Ni–8Sn–0.3Si–0.1Ti alloy. In addition, the tensile strength and elongation of this alloy reach 1117 MPa and 16.4% after aging at 400 °C for 240 min.

#### 4.3.2. Solid Solution + Cold Deformation + Aging

**Figure 12:**
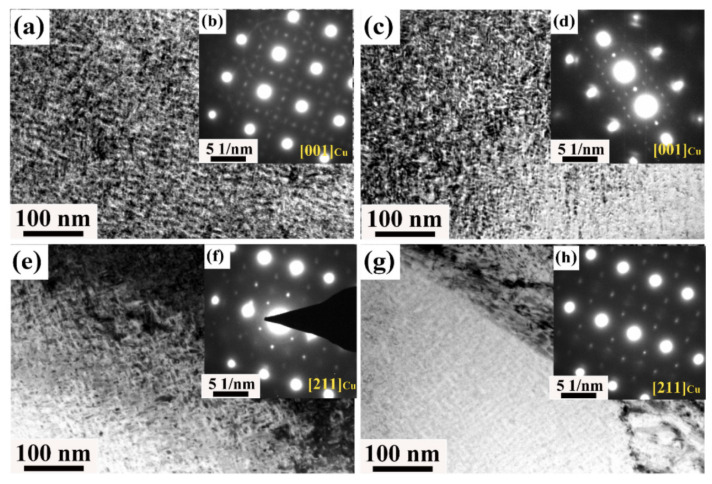
TEM images and SADP patterns of (**a**,**b**) 30%-cold-deformed Cu–15Ni–8Sn alloy; (**c**,**d**) 30%-cold-deformed Cu–15Ni–8Sn–0.2P alloy; (**e**,**f**) 50%-cold deformed Cu–15Ni–8Sn alloy; (**g**,**h**) 50%-cold-deformed Cu–15Ni–8Sn–0.2P alloy. (Reproduced with permission from Elsevier [59]).

By applying cold deformation between solution and aging, the proper amount of dislocations and deformed microstructure can be formed in the copper matrix. This can reserve energy and provide channels for precipitation in the subsequent aging process. This effect is beneficial to improving the precipitation rate and precipitation efficiency and further improving the comprehensive properties of the alloy. For example, Guo C. et al. [59] studied the effect of cold deformation (30%, 50%, 70%, and 90%) on the microstructure and properties of Cu–15Ni–8Sn(P) alloy. The results show that cold deformation can inhibit the transformation from the DO_22_-ordered phase to the L1_2_-ordered phase (Figure 12). In addition, the tensile strengths of Cu–15Ni–8Sn and Cu–15Ni–8Sn–0.2P alloys in peak aging states are 1245 MPa and 1303 MPa, respectively, by applying 70% cold deformation before aging. However, Lefevre B. G. et al. found that [6] both the hardening and discontinuous transformation growth rates are increased by extensive deformation prior to aging. Prior deformation decreases the time to peak strength but does not significantly affect the peak strength increment.

#### 4.3.3. Multistage Combined Thermomechanical Treatment

**Figure 13:**
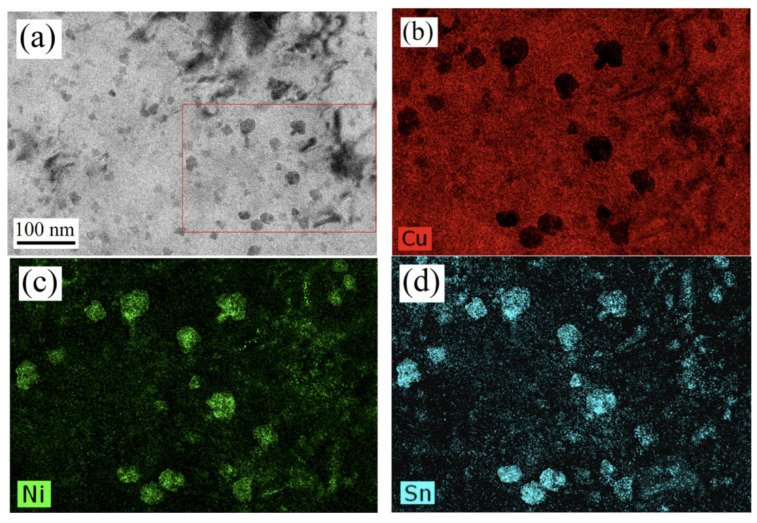
HAADF-STEM image and element maps of the Cu–15Ni–8Sn alloy compressed with 5 × 10^−4^ s^−1^. (**a**) image of particles, and the SEM-EDS test is carried out in the red box area; (**b**) element map of Cu; (**c**) elements map of Ni; (**d**) element map of Sn. (Reproduced with permission from Elsevier [60]).

Peng G. et al. [60] investigated the microstructure and subsequent re-aging behavior of a Cu–15Ni–8Sn alloy pre-deformed with dynamic strain aging (DSA). The strain rates selected in this article are 5 × 10^−5^ s^−1^, 5 × 10^−4^ s^−1^_,_ and 5 × 10^−2^ s^−1^, and the compression deformation is 25%, 50%, and 75%, respectively. Finally, some typical specimens are selected to be re-aged in the salt bath at 400 °C at different times. The results show that DSA has an inhibitory effect on spinodal decomposition and results in the segregation and clustering of Sn and Ni (as shown in Figure 13), eventually leading to the precipitation of Ni_3_Sn. As the aging time prolongs, the Ni_3_Sn phase grows and coarsens, finally transforming into a cellular precipitate of γ (DO_3_) phase. In addition, DSA pretreatment can significantly shorten the peak aging time of the re-aging process. Jiang Y. et al. [55] studied the effect of two-stage thermomechanical treatment (pre-aging at 400 °C for 30 min, cold rolling with 60% reduction, and aging at 450 °C for different times) on the microstructure and properties of Cu–15Ni–8Sn–1.0Zn–0.5Al–0.2Si alloy. Compared with single-stage deformation aging treatment (cold rolling with 60% reduction and aging at 450 °C for different times), the peak-aged alloy treated with two-stage thermomechanical processing shows a tensile strength of 1176 MPa, elongation of 3.86%, and a strength–ductility product of 4539 MPa%. The high strength of the studied alloy is mainly attributed to the combined effects of precipitation strengthening and substructure strengthening. The interaction between the nascent nanoparticles that form during pre-aging and the dislocation configurations that form during cold rolling promotes precipitation in the matrix. It suppresses the formation of cellular precipitates and coarse precipitates at grain boundaries, improving the comprehensive properties of the alloy.

## 5. Application Prospect and Development Trend

The Cu–15Ni–8Sn alloy can be used in service conditions up to 250 °C, and its stress relaxation resistance is better than that of beryllium bronze. It is one of the most promising materials to replace beryllium bronze alloy in the field of electronic equipment and instruments. In recent years, with the in-depth study of this alloy by scholars at home and abroad, the requirements for the comprehensive properties of this alloy have been constantly improving. For example, on the basis of meeting high strength, it is required to develop in the coordinated control direction of high toughness, high wear resistance, high corrosion resistance, and high electrical conductivity. This greatly broadens the application prospects of the Cu–15Ni–8Sn alloy in aerospace, heavy-duty equipment, oil and gas exploitation, ocean engineering, the electronic industry, and other fields. Therefore, the author believes that the future development direction of the Cu–15Ni–8Sn alloy in the process of development and application is as follows:

(1) The multi-component synergistic strengthening effect of microalloying in Cu–15Ni–8Sn alloy should be further exerted. The inhibition mechanism of element interaction on segregation in Cu–15Ni–8Sn alloys should be further clarified. The synergistic improvement mechanisms for strength, toughness, wear resistance, and corrosion resistance of alloys should be further revealed. Based on the genetic engineering of materials, the composition design basis of high-performance Cu–15Ni–8Sn alloy should be further put forward, and the database should be established.

(2) The effect of the heredity of the solidification structure on the microstructure and properties of the Cu–15Ni–8Sn alloy during subsequent deformation and heat treatment should be given more attention. The application of in situ observation in the study of amplitude-modulated decomposition and discontinuous precipitation behavior of Cu–15Ni–8Sn alloys should also receive more attention. Furthermore, through material computation and experimental verification, the phase transformation sequence of this alloy can be deeply and intuitively revealed. It is necessary to further clarify the regulating mechanism of microstructure characteristics on the properties of alloys, such as high strength and toughness, high wear resistance, and high corrosion resistance. Furthermore, it is expected to further optimize and improve the industrial production process of Cu–15Ni–8Sn alloy.

(3) New application scenarios and new performance requirements for Cu–15Ni–8Sn alloys should be given more attention. Cu-15Ni-8Sn alloy is required not only to have high strength, but also to have excellent corrosion resistance, wear resistance, and stress relaxation resistance. For example, spherical bearings for aerospace use require significantly higher strength than those used in other applications due to load concentration. In addition, with the development of service conditions for key components in deep-sea oil and gas exploration, underground oil and gas development, and other fields involving high temperatures, high pressure, and complex corrosive media, there is a growing need for materials with enhanced performance and durability. As new energy vehicles and the renewable energy industry bloom, Cu–15Ni–8Sn alloy has great application potential because of its excellent properties. Furthermore, the development of new technologies further promotes the demand for Cu–15Ni–8Sn series new materials with high strength, toughness, and wear resistance.

## Figures and Tables

**Table 1:** Properties of Cu–15Ni–8Sn alloy products prepared by Materion Company [9].

Alloys	Tensile Strength (MPa)	0.2% Offset Yield Strength (MPa)	Elongation (%)	CVN Impact Strength (J)	K_1c_ Fracture Toughness (MPa·m^1/2^)
UNS C72900 ToughMet^®^3AT	760–930	620–760	4.0–15	5.0–12	33–49
UNS 72900 ToughMet^®^3TS	730–1140	655–1035	3.0–18	5.0–40	44–77
UNS C72900 BrushForm^®^158	655–1415	515–1380	1.0–22	-	-

**Table 2:** Properties of Cu–15Ni–8Sn alloy products from Lebronze Company. (The data were collected from the company website [16]).

Alloys	Tensile Strength (MPa)	0.2% Offset Yield Strength (MPa)	Elongation (%)	Hardness (HRC)
HARDIALL^®^ AMS 4596 C72900	876–910	738–745	3–10	30
HARDIALL^®^ AMS 4597 C72900	1075–1137	1020–1069	3–6	34–35
HARDIALL^®^ TS 120 U C72900	825	755	15	22–24
HARDIALL^®^ TS 160 U C72900	1100–1105	1020–1035	3	32–34
HARDIALL^®^ TS 95 C72900	725–730	655	18	15
HARDIALL^®^ TX 110 C72900	875–910	760	6–10	30
HARDIALL^®^ TX 90 C72900	760	620	15	26

**Table 3:** Comparison of the strength and elongation ratio of the Cu–15Ni–8Sn–*X* alloy with different trace alloy elements.

Alloys (wt.%)	Tensile Strength (MPa)	Elongation (%)	Reference
Cu–15Ni–8Sn–0.2Nb	1400	1	[37]
Cu–15Ni–8Sn–0.21Nb	702.9	18.4	[39]
Cu–15Ni–8Sn–0.2Nb	653	17	[30]
Cu–15Ni–8Sn–0.3Nb	1030	9.1	[18]
Cu–15Ni–8Sn–0.3Nb	943	5.5	[40]
Cu–15Ni–8Sn–0.4V	990	-	[41]
Cu–15Ni–8Sn–0.1Fe	1250	6.45	[44]
Cu–15Ni–8Sn–0.5Co	1250	-	[45]
Cu–15Ni–8Sn–0.3Si	795	31.2	[36]
Cu–15Ni–8Sn–0.2Ti	860.36	-	[47]
Cu–15Ni–8Sn–0.3Ti	1024	17.9	[48]
Cu–15Ni–8Sn–0.1Ti	1088	17.3	[49]
Cu–15Ni–8Sn–0.3Ti	875	24.1	[50]
Cu–15Ni–8Sn–0.5Zr	980	-	[51]
Cu–15Ni–8Sn–0.2P	1303	-	[52]
Cu–15Ni–8Sn–0.2Y	1166	-	[53]
Cu–15Ni–8Sn–0.3Si–0.1Ti	910	30	[50]
	1117	16.4	
Cu–15Ni–8Sn–0.25Mn–0.1Fe	1268	3.6	[54]
Cu–15Ni–8Sn–1.0Zn–0.5Al–0.2Si	1176	3.86	[55]
Cu–15Ni–8Sn–0.14Fe–0.11Mn–0.22Zn	855	15.2	[56]

**Table 4:** Comparison of properties of Cu–15Ni–8Sn alloys after heat treatment by different processes.

Alloy System	Preparation Process	Heat Treatment Process and Parameters	Tensile Strength (MPa)	Hardness (HV)	Elongation (%)	References
Cu–15Ni–8Sn	Solution + Aging	Solution: 850 °C/6 h Aging: 400° C/4 h	890	-	-	[57]
Cu–15Ni–8Sn	Solid solution in two-phase region + Aging	Solution: 725 °C/4 h Aging: 425 °C × 8 h	889	-	-	[58]
Cu–15Ni–8Sn–0.3Si–0.1Ti	Solution + Aging	Solution: 820 °C/1 h Aging: 400 °C/4 h	1117	-	16.4	[50]
Cu–15Ni–8Sn–0.2P	Solution + Cold deformation + Aging	Solution: 850 °C × 15 h Cold deformation: 70% Aging: 400 °C/1 h	1303	-	-	[59]
Cu–15Ni–8Sn	Solution + Dynamic strain aging (DSA) + Aging	Solution: 850 °C/1 h DSA: 250 °C, 75%, 5 × 10^−5^ s^−1^ Aging: 400 °C/10 min	-	390	-	[60]
Cu–15Ni–8Sn–1.0Zn–0.8Al–0.2Si	Two-stage homogenization annealing (THA) + Solution + Cold deformation + Aging	THA:830 °C/2 h + 850 °C/2 h Solution: 850 °C/1 h Cold deformation: 60% Aging: 450 °C/0.5 h	1144	-	3.29	[61]
Cu–15Ni–8Sn–1.0Zn–0.5Al–0.2Si	Two-stage homogenization annealing(THA) + Hot rolling + Solution + Pre-aging + Cold deformation + Aging	THA: 830 °C/2 h + 850 °C/2 h Hot rolling: 850 °C, 70% Solution: 850 °C/1 h Pre-aging: 400 °C/0.5 h Cold deformation: 60% Aging: 450 °C/0.5 h	1176	-	3.86	[55]

## Data Availability

No new data were created.
